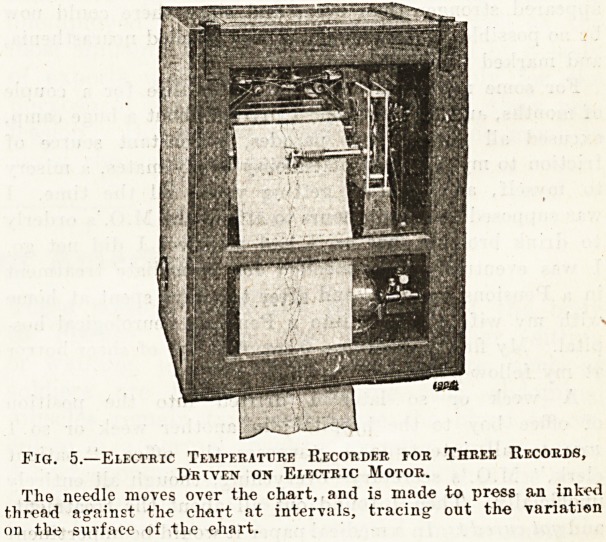# Electrical Thermometers and Boiler Temperatures

**Published:** 1921-06-18

**Authors:** 


### HOSPITAL ENGINEERING REFORMS.

### III.—Electrical Thermometers and Boiler Temperatures.

Electrical thermometers, or pyrometers, are made on two different principles. In one the variation in the electrical resistance of a length of platinum wire, due to changes in the temperature to which the platinum wire is subject, is made to cause the motion of the needle of a galvanometer over a dial, which is graduated in degrees F. or C., as may be required. In the other, the fact is made use of that when the junction of two dissimilar metals is exposed to heat an electrical pressure is set up at the junction; this furnishes an electric current whose strength may be measured by a suitably arranged galvanometer.

Fig. 3 shows one kind of resistance thermometer; a platinum wire is enclosed inside the tube shown at the bottom of the figure, and the tube is screwed into a cavity provided for it by means of the thread shown. The ends of the platinum wire are connected to terminals provided outside, as shown, and these are led to the instrument forming the Wheatstone's Bridge. A small battery, whose pressure has to be maintained constant, is also connected to the apparatus when it is being used, and the temperature to which the platinum wire is exposed is reflected in the galvanometer, whose needle moves over a graduated dial, forward or backward, according to the rise or fall of the temperature. The galvanometer, or the indicator, as it is usually called, is made in different forms; a convenient one is that shown in fig. 4, which is arranged to be fixed upon a wall, where it can be read quite easily by the boiler attendants.

Fig. 3.—A Platinum Resistance Thermometer.

The platinum wire is enclosed inside the tube shown, and is connected to the terminal screws shown at the top.

One indicator is alone necessary, unless a recorder is required, to show the temperature at several points in the boiler system. The connecting wires from the different thermometers are led to a switchboard, usually fixed under the indicator; and it is arranged, sometimes by plug switches, and sometimes by other forms, to connect any particular thermometer to the indicator at any moment. The boiler attendant, or the engineers-in-charge, can read off the temperatures at the different thermometer stations in a very short time, by plugging in, or switching on each one in succession.

The electric resistance thermometer is also made to record the temperatures for any time that may be required, and for any number of thermometer stations. The usual arrangement consists of a drum, carrying a chart ruled in the usual way, and made to revolve, either by clockwork or by an electric motor; in either case the revolution of the drum occupies a fixed time. In place of the needle sweeping over the dial of a galvanometer, a pointer is arranged to be depressed at intervals on to a thread impregnated with ink, which marks the surface of the chart. There may be several threads marking the temperature for several thermometers at the same time, or any particular thermometer may be connected to its own recorder. Fig. 5 shows another form of chart, ruled in the same way, but arranged to move forward continuously as the drum revolves, a given length of chart corresponding to a certain time. The form shown is worked by an electric motor.

The electric resistance thermometer is made for temperatures ranging from very low figures, a long way below zero, up to 1,000° F. They can be employed for reading the temperature at the base of the chimney, at the entrance to the economiser, where one is fixed in connection with the boiler; the economiser will be described later in these articles. It can also be used in certain parts of the flues, or the corresponding spaces in water-tube boilers, through which the hot flue gases pass. The hot gases, without an economiser, are usually delivered at the base of the chimney at 600° F., or thereabouts; with an economiser, the temperature at the chimney should be 300° F., and 600° F. where the hot gases enter the economiser. The temperature rises along the path of the hot gases towards the furnace, and it will be found to help the economic working of the boiler to measure these temperatures at different points periodically. Measuring them at different points in the flues will show whether air is leaking through the brickwork, and so diluting the gases and lowering the efficiency of the boiler as a whole. For higher temperatures than 1,000° F. the thermo-electric thermometer, or pyrometer, is employed; it will be described in the next article.

Fig. 4.—The Indicator, or Galvanometer.
Used with electric thermometers. This pattern is arranged to be fixed on a wall; the needle moves round the scale, as will be seen.

Fig. 5.—Electric Temperature Recorder for Three Records, Driven on Electric Motor.
The needle moves over the chart, and is made to press an inked thread against the chart at intervals, tracing out the variation on the surface of the chart.

## Figures and Tables

**Fig. 3. f1:**
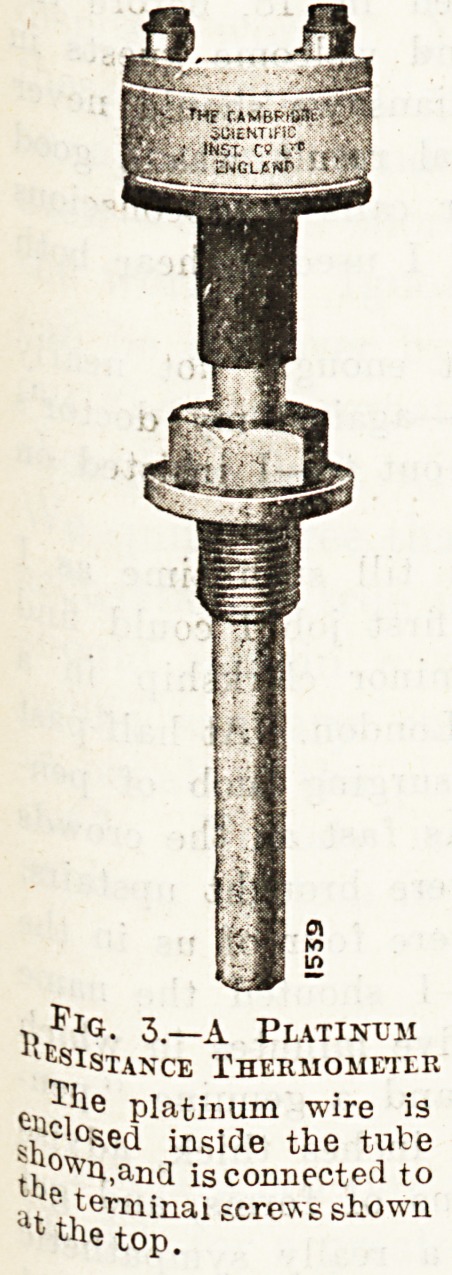


**Fig. 4. f2:**
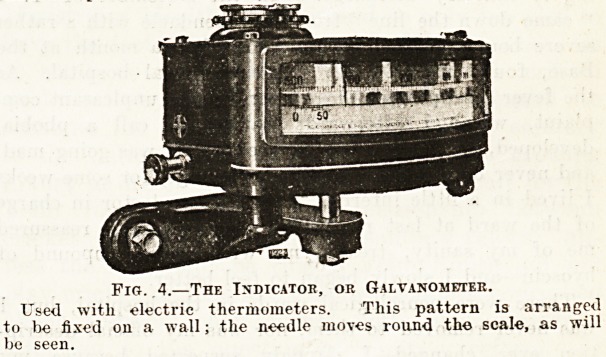


**Fig. 5. f3:**